# Review of the Recent Advances in Electrospun Nanofibers Applications in Water Purification

**DOI:** 10.3390/polym14081594

**Published:** 2022-04-14

**Authors:** AbdElAziz A. Nayl, Ahmed I. Abd-Elhamid, Nasser S. Awwad, Mohamed A. Abdelgawad, Jinglei Wu, Xiumei Mo, Sobhi M. Gomha, Ashraf A. Aly, Stefan Bräse

**Affiliations:** 1 Department of Chemistry, College of Science, Jouf University, Sakaka 72341, Al Jouf, Saudi Arabia; 2 Composites and Nanostructured Materials Research Department, Advanced Technology and New Materials Research Institute, City of Scientific Research and Technological Applications (SRTA-City), New Borg Al-Arab 21934, Egypt; ahm_ch_ibr@yahoo.com; 3 Research Center for Advanced Materials Science (RCAMS), King Khalid University, Abha 61413, Asir, Saudi Arabia; aawwad@kku.edu.sa; 4 Department of Pharmaceutical Chemistry, College of Pharmacy, Jouf University, Sakaka 72341, Al Jouf, Saudi Arabia; mhmdgwd@ju.edu.sa; 5 Key Laboratory of Science and Technology of Eco-Textile, Ministry of Education, College of Chemistry, Chemical Engineering and Biotechnology, Donghua University, Shanghai 201620, China; jw@dhu.edu.cn (J.W.); xmm@dhu.edu.cn (X.M.); 6 Chemistry Department, Faculty of Science, Cairo University, Giza 12613, Egypt; smgomha@iu.edu.sa; 7 Chemistry Department, Faculty of Science, Islamic University of Madinah, Madinah 42351, Al Jamiah, Saudi Arabia; 8 Chemistry Department, Faculty of Science, Organic Division, Minia University, El-Minia 61519, Egypt; ashrafaly63@yahoo.com; 9 Institute of Organic Chemistry (IOC), Karlsruhe Institute of Technology (KIT), Fritz-Haber-Weg 6, 76133 Karlsruhe, Germany; 10 Institute of Biological and Chemical Systems-Functional Molecular Systems (IBCS-FMS), Director Hermann-von-Helmholtz-Platz 1, 76344 Eggenstein-Leopoldshafen, Germany

**Keywords:** electrospinning, nanofibers, wastewater treatment, polymers, organic and inorganic pollutants, oil/water (O/W) separation

## Abstract

Recently, nanofibers have come to be considered one of the sustainable routes with enormous applicability in different fields, such as wastewater treatment. Electrospun nanofibers can be fabricated from various materials, such as synthetic and natural polymers, and contribute to the synthesis of novel nanomaterials and nanocomposites. Therefore, they have promising properties, such as an interconnected porous structure, light weight, high porosity, and large surface area, and are easily modified with other polymeric materials or nanomaterials to enhance their suitability for specific applications. As such, this review surveys recent progress made in the use of electrospun nanofibers to purify polluted water, wherein the distinctive characteristics of this type of nanofiber are essential when using them to remove organic and inorganic pollutants from wastewater, as well as for oil/water (O/W) separation.

## 1. Introduction

Recently, due to overpopulation, urbanization, increases in different industrial and human activities, landfill expansion, mining activities, and urban wastewater, various environmental problems related to water pollution are becoming more worrying and critical [1,2]. Huge amounts of different toxic pollutants are discharged daily into water resources, causing increasingly severe water pollution problems, contaminating aquatic bodies, and damaging the environment [2,3]. Therefore, water resources are tainted with various pollutants, such as organic and inorganic pollutants, pharmaceutical wastes, nanoparticles, pathogens, microbes, oil, and other harmful materials [4]. Dyes and heavy metal ions are considered major water pollutants [4]. At present, water contamination is one of the most serious global issues due to the scarcity of water sources. To remediate these serious circumstances, different purification techniques using novel fabricated materials have been developed to remove harmful wastewater contaminants [1,2]. Recently, nanotechnologies and nanomaterial sciences have attracted much attention, with a focus on fabricating nanomaterials with unique properties for use in various water purification applications. Electrospinning is one of the most important techniques that can be used to fabricate such nanomaterials. It is an advanced continuous fiber-generating technique that produces ultra-small fibers ranging from the nano- to the microscale. In addition, it is a versatile technique, is cost-effective, and is easily accessible [5]. Typically, an electrostatic force is applied across a set distance between a syringe containing a polymer solution and a collector, creating a sufficiently high electric field to overcome the polymeric material’s surface tension. Thus, the polymeric solution will be converted into a fiber structure that is deposited on the collector, forming a nonwoven mat with an interconnected porous structure, high surface area, high porosity, and light weight.

Electrospun nanofiber mats can be modified to meet the needs of certain applications, such as by blending functional additives into the electrospinning solution [6], incorporating an active species, surface coating, or interfacial polymerization (IP) [7,8,9]. These modification techniques imbue electrospun nanofibers with strong advantages for applications in different fields, such as water purification. In addition to these superior characteristics, the electrospun nanofibers used in wastewater treatment must be water-insoluble, safe, and easily recovered.

Many researchers have studied the applications of electrospun nanofibers, and addressed topics related to specific fields, such as the purification and treatment of wastewater [2,6,7,8,9,10,11,12,13,14,15,16], biomedical applications, drug delivery [17,18,19,20,21,22,23,24,25,26], oxygen electrocatalysis [27], water splitting [28], intelligent food packaging [29], sustainable applications [30], solid-phase extraction in liquid chromatography [31], and electronical and energy applications [32,33,34].

Recent reviews have discussed the effective removal of various pollutants using nanomaterials, and outlined solutions to water challenges [6]. Additionally, recent advances in the application of polymeric electrospun nanofibers mats to remove different pollutants from aqueous systems via membrane separation techniques, and the effects of morphology of the nanofibers, were also addressed [7]. Many other reviews have provided a broad overview of water purification using various materials and techniques. On the other hand, works outlining the applications of different types of electrospun nanofibers in various water purification systems over the last five years are relatively limited. Therefore, this review provides a comprehensive overview of recent applications of electrospun nanofibers to treat water contaminated with different pollutants, such as organic and inorganic pollutants, and to perform oil/water (O/W) separation using different techniques. We address the removal of different types of organic pollutants and different types of metal ions (such as lanthanide and actinide) and heavy metal ions, and O/W separation using different electrospun nanofiber materials. Additionally, the recently fabricated sustainable nanofibers (eco-friendly) used in water purification processes are discussed. Finally, the recent achievements, challenges, and future perspectives are outlined.

## 2. Some Types of Water Pollutants

### 2.1. Organic Pollutants

Organic contaminants in water, such as dyes, phenols, detergents, pharmaceuticals, and others, can be discharged from the domestic sewage, agriculture, food, paper, and leather industries. Such wastes require a high amount of oxygen to complete the oxidative decomposition process, which decreases the quantity of dissolved oxygen in the water, thereby threatening the lives of aquatic organisms and ecosystems [4,35].

When some dyes are introduced to surface water, they produce degradable byproducts that can have carcinogenic effects, and most of them have hydrophobic properties, accumulate in the water, and can penetrate the tissues of some aquatic organisms and humans. Moreover, dyes are extremely toxic pollutants, and are discharged from various industries, such as plastics, rubber, and textile, into the ecosystem and water resources [36]. The effluent of such materials into the water results in serious harm to humans. Other more harmful effects of the dyes include preventing light from penetrating water, which inhibits photosynthesis in aquatic plants. Removing dyes from colored water is a vital issue. This is because the presence of dye in water, even at low concentrations, will prevent sunlight penetration into the water. Consequently, the lives of aquatic organisms will be put at risk. Moreover, the presence of the dyes in drinking water may have carcinogenic effects [37].

### 2.2. Inorganic Pollutants

One of the most dangerous side effects of the rapid growth of many industries, such as electroplating, tanneries, battery manufacturing, electroplating, etc., is the discharging of effluents contaminated with toxic heavy metal ions into water bodies [38]. Even at very low concentrations, the presence of these contaminants in the aquasphere impacts all living organisms, humans, animals, and plants. Heavy metals have carcinogenic and toxic effects, which may cause adverse ecological and human health effects. Some metal cations cause damage to humans’ central nervous systems and kidneys [38]. Exposure to these metal ions, even at trace levels, is believed to pose risks to human, animal, and aquatic organisms. In addition to the increasing water scarcity problems, removing such contaminants from water is a pressing need, and remains important for scientists.

### 2.3. Oil Pollution

The processing of natural oil is the main source of crude oil and its derivatives. Leakage and spill accidents occurring during the utilization and transportation of oil [39] have resulted in polluted water and soil, which hurts living beings. Numerous separation techniques have been applied to separate oil and water, such as in situ burning, filtration, centrifugation, neutralization, etc.; these techniques suffer from some disadvantages, such as low efficiency, long time requirements, high costs or causing secondary pollution. Moreover, the oil remaining in water after poor separation still poses risks to human health and aquatic life. Consequently, efficient and eco-friendly materials and treatment techniques require immediate investigation by governments and researchers [1]. With the increasing the knowledge of the fabrication and processing of nanostructured materials, scientists are now investigating and developing various types of novel polymeric nanomaterials that have good physicochemical characteristics and can be used in different applications, using advanced techniques; in this way, new technologies have been established [1,2,3].

## 3. Nanotechnology and Water Treatment

Since the beginning of this century, nanotechnology and nanostructured materials have attracted considerable attention in most research areas due to their unique properties. Nanomaterials are described as materials that are <100 nm in at least one dimension. The chemical and physical properties of materials at this size are greatly improved due to their less bulky size. For example, the density of binding sites at the nanoscale is attributed to their high surface area. Moreover, nanomaterials provide enhanced surface free energy, thus increasing the materials’ surface reactivity. These advantages make nanomaterials useful in water treatment applications [40]. Among the currently available nanostructured materials, nanofibers have illustrated unique capabilities and excellent characteristics that enable them to overcome the limitations of conventional fibrous structures [41]. The prepared nanofibers, especially those fabricated by electrospinning techniques with large surface areas, tunable porosity, good mechanical characteristics, uniform pore distributions, and good functional capabilities, play significant roles in water treatment [40,42,43].

## 4. Applications of Electrospinning Nanofibers in Wastewater Treatment

Many pollutants have been discharged into freshwater supplies and their surrounding ecosystems, including organic and inorganic pollutants. Oil spills have serious impacts on ecosystems and human health [44,45,46]. Therefore, nanofibrous mats can be easily modified by blending functional additives into an electrospinning solution, incorporating an active species, and applying the surface coating via various water treatment processes. These modifications impart advantages on electrospun nanofibers used for water/wastewater treatment applications. As such, much effort has been directed towards developing new technologies and materials, such as electrospinning nanofibers, to reduce such dangerous pollutants, particularly as the world is suffering a water crisis related to the scarcity of conventional freshwater reservoirs [45,47].

## 5. Electrospun Nanofibers for Water Treatment

Recently, the demand for high-quality water has increased dramatically worldwide [10,48,49,50,51,52,53]. Consequently, many works have focused on decontaminating wastewater discharged from various sources into rivers, reservoirs, or streams [10]. As such, scientists are attempting to find and develop alternative, cost-effective, sustainable technologies to treat or recycle wastewater [12]. By 2025, about 3.5 billion people around the world will face freshwater shortages. Heavy metals ions are the main class that has great physiological impacts on health and ecosystems. Additionally, organic pollutants at lower concentrations have high toxicity effects on human health and the environment. Therefore, water purification issues have become more pressing [54]. Therefore, the development of efficient and advanced filtration and separation techniques in order to overcome water purification challenges is becoming an urgent need. Due to the unique properties and potential advantages of the fabricated electrospun nanofibers mats, they have been considered as suitable platforms to combine filtration and adsorption techniques—with considerable purification efficiency—to remove organic pollutants and heavy metal ions from contaminated water. On the other hand, nanofiber membranes must have many contact sites in order to be an ideal material for removing such pollutants. Consequently, the fabrication of novel nanofibers structures has gained great attention from researchers. When electrospun nanofibers are utilized in water purification processes as ultrafiltration membranes, the adsorption process, via electrostatic interactions, ion exchange, or chelation, is expected to be the major pollutant-removal mechanism. Furthermore, if electrospun nanofibers are used as NF/RO membranes, the issue of size-based rejection must be considered.

Therefore, nanofibrous structures fabricated by electrospinning methods play a considerable role, and have shown significant potential for use in wastewater treatment due to their favorable characteristics related to removing the organic and inorganic pollutants from aqueous media [2,10,52,53,54]. In addition, harnessing such innovative techniques and scalable routes to fabricate novel materials may reduce society’s reliance on traditional purification technologies, and decrease the consumption of materials and energy by these technologies [53].

### 5.1. Removal of Dyes and Organic Pollutants

**Figure 1:**
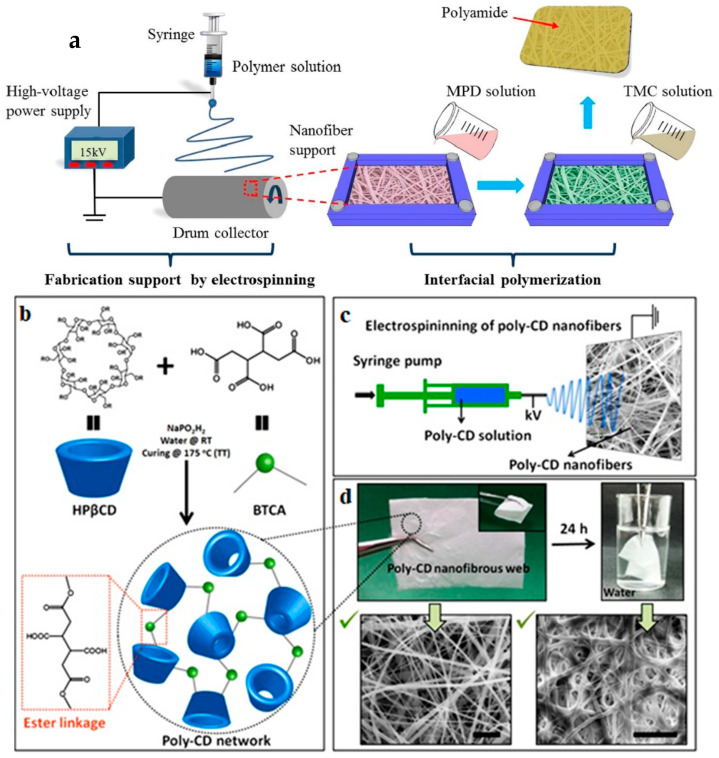
Schematic illustration of the fabrication (**a**) of electrospun nanofiber-supported TFC membrane, reproduced with permission of Elsevier [55]. (**b**–**d**) of the crosslinked poly-CD nanofibrous web, reproduced with permission of Springer Nature [56].

Various novel and promising nanofibers and their potential modifications have been assessed for their use in removing different organic pollutants from wastewater. Self-sustained electrospun polyacrylonitrile (PAN) nanofiber-supported polyamide (PA) thin-film composite (PA/PAN-eTFC) membranes are one such material that was prepared and tested for the removal of tetracycline (TC) from wastewater. Initially, the support for the PAN nanofiber was synthesized by electrospinning (Figure 1a). After that, the obtained PAN nanofiber mat was laminated with a paper laminator. The laminated nanofiber support exhibited hydrophilicity and mechanical features, with a water contact angle of 32.3 ± 1.3°, stress of 13 ± 0.77 MPa, and strain of 68 ± 0.28%, respectively. The polyamide composite mat prepared thereon showed low structural parameters (S = 168 μm) and enhanced permselectivity (A = 1.47 LMH bar−1, B = 0.278 LMH), and reached >57 LMH water flux when utilizing 2.0 M sodium chloride as the draw solution. The fabricated PA/PAN-eTFC membranes achieved a high rejection of TC (>99.9%), and 15–22% water recovery was reached after seven hours using the FO–MD hybrid method [55]. Figure 1a shows a diagram of the fabrication of the electrospun nanofiber-supported TFC membranes [55], and the fabrication of the crosslinked poly-CD nanofibrous web [56].

An electrospinning approach was employed to prepare a highly efficient molecular filter membrane composed of crosslinked insoluble poly-CD nanofibers, and this achieved the excellent and rapid uptake of organic pollutants from a liquid environment [56]. Table 1 summarizes the optimum conditions for some of the nanofibers used to remove many types of organic and inorganic pollutants [55,56,57,58,59,60,61,62,63,64,65,66,67,68,69,70,71,72,73,74,75,76,77,78,79,80,81,82,83,84,85,86,87,88,89,90,91,92].

With the rapid development of dye-related industries, huge amounts of wastewater are continuously discharged into surrounding environments. These dyes, such as methylene blue (MB), may be degraded, and produce carcinogens and toxic products. These pollutants are dangerous, and can adversely affect the environment and human health. Thus, novel nanofibers have been developed to eliminate such pollutants from water resources [57,58,59,60,61,62,66,67,69,70,71,80,81,82]. A deacetylated cellulose acetate (DA)@polydopamine (PDA) composite nanofiber membrane was prepared using electrospinning and surface functionalization, and applied to adsorb MB dye from an aqueous media. The results show that a uniform coating layer of PDA was successfully generated on the DA nanofibers’ surface.

Moreover, according to the Langmuir isothermal model, the DA@PDA nanofiber membrane possessed an MB adsorption capacity of 88.2 mg/g, which was 8.6-fold greater than DA nanofibers [57]. Additionally, polyaniline nanofibers grafted with phytic acid (DPANI) were employed for the adsorption of MB dye. The analysis indicates that DPANI possessed a superior adsorption capacity compared to ungrafted polyaniline nanofibers, as it has six phosphoric groups on its surface, which improves the attachment of MB [58]. Methylene blue was removed using poly(methylmethacrylate) nanofiber membranes covered with reduced graphene oxide (PMMA-rGO), prepared by solution blow-spinning (SBS). After that, the obtained nanofibers were adjusted in three steps: (i) the plasma treatment of the poly(methylmethacrylate) surface to generate oxygen functional groups on the PMMA nanofiber’s surface; (ii) GO adsorption, which occurs on the previously generated hydrophilic groups; and (iii) GO reduction, carried out by the chemical reduction of graphene oxide to form a stable rGO layer, involving the loss/conversion of surface O-functional groups and the partial recovery of the π-electron structure. This last step had a significant impact on the removal efficiency of the composite.

Further, a π–π stacking attachment significantly helps to remove MB, with a maximum adsorption capacity of 698.51 mg g^−1^ [59]. Using the SBS technique, a porous N-carbon/silica nanofiber (PN-CSN) was prepared by combining polyvinylpyrrolidone (PVP), tetraethyl orthosilicate (TEOS), and ethanol. Subsequently, we performed the carbonization of the fiber samples at 550 °C in air, and evaluated the removal of MB. According to the Langmuir model, the fabricated nanofiber composite had an enhanced adsorption capacity (400 mg g^−1^) in relation to MB-dye [60]. A novel poly(vinyl alcohol) (PVA)/starch hydrogel nanofiber membrane was prepared in water through the electrospinning approach, followed by thermal crosslinking, and this was applied to remove (MB) from the aqueous media. According to the Langmuir isotherm model, the nanofiber can adsorb an MB dye at 400 mg g^−1^. Moreover, the mat could be regenerated using a methanol solution containing 5% (*v*/*v*) HCl [61].

An electrospun cellulose acetate poly(ethylene oxide) nanofiber membrane loaded with bacterial cells (Bacillus paramycoides) was able to remove MB from aqueous media through a combination of adsorption and biodegradation strategies. Furthermore, the membrane could be successfully reused, and 44% of the MB removal capacity can still be obtained after the fourth usage, by which 93% of the MB could be removed within 48 h [62].

At present, the synthesis of MOF-based bionanomaterials plays a vital role in wastewater treatment technologies. Consequently, many works have attempted to fabricate such electrospun nanofibers. A zeolitic imidazolate framework-8 (ZIF-8) crystal was anchored on electrospun nanofiber chitosan/polyvinyl alcohol (Chi/PVA-ENF) to fabricate ZIF-8@Chi/PVA-ENF, and this was used to adsorb Malachite green (MG) from aqueous media. Multiple cycles were applied to coat the ZIF-8 onto the Chi/PVA-ENF in the first (ZIF-8@Chi/PVA-ENF(1)), second (ZIF-8@Chi/PVA-ENF(2)), and third cycles (ZIF-8@Chi/PVA-ENF(3)). The experimental data show that the ZIF-8@Chi/PVA-ENF(2) achieved the best Langmuir adsorption capacity (1000 mg/g) compared to the other composites. Moreover, recycling tests have demonstrated that ZIF-8@Chi/PVA-ENF(2) has the highest chemical stability [63].

Additionally, a Mo_2_N/MoO_2_ composite nanofiber has been made using electrospinning and a regulated nitridation process. The composite nanofibers can achieve the efficient absorption of Rhodamine B (RhB). Furthermore, the absorption activity of the nanofibers remains stable even after it is recycled in four runs [64]. An electrospinning strategy has been used to fabricate Faujasite zeolite (FAU)/poly(lactic acid) (PLA) nanocomposite nanofibers, to be used in the adsorption of dyes from wastewater. The FAU particle concentrations, derived from poly(lactic acid), and the flow rates were evaluated upon the nanofibers’ fabrication. The adsorption analysis has revealed that this composite is an efficient material for the removal of cationic dyes ((RB) and (MB)) from wastewater [65]. In addition, the trifunctional polyurethane (PU)/graphene oxide (GO) electrospun membrane (PU/GO) has yielded many results pertaining to the removal of both RB and MB [66].

Moreover, the fabricated membrane exhibited antifouling and bacteria growth inhibition abilities, and it also showed antibacterial capacities against Gram-negative and Gram-positive bacteria. Additionally, the superhydrophobic PU/10GO membrane illustrated a large water flux of 17,706 Lm^−2^h^−1^, and it also showed excellent antifouling behavior when used in separating oil-in-water emulsions, with a separation efficiency of 99.99% [66].

Various nanofibrous mats have been fabricated and applied for dye elimination in aqueous media. The zeolitic imidazolate framework (ZIF-8)/polyacrylonitrile (PAN) nanofibrous mats are an example [67]. In this work, 2-methylimidazole (2-MI) was mixed into a polyacrylonitrile (PAN) polymeric solution for electrospinning. After this, zeolitic imidazolate framework (ZIF-8) nanocrystals were grown on the PAN nanofibers’ surface through pre-treatment, to form (ZIF-8/PAN) nanofibrous mats. The findings reveal that the ZIF-8/PAN nanofibrous mats showed good adsorption capacities as well as reusability [67]. Additionally, a water-stable and eco-friendly membrane was prepared from carboxymethyl cellulose (CMC) (which serves as an anionic adsorbent) and cellulose nanofibrils (CNFs) (as the filler) by the solvent and unidirectional freeze-casting strategy, and using citric acid (CA) as the crosslink to remove cationic dyes. The obtained freeze-casted membrane showed an anisotropic to isotropic and highly (>90%) porous network structure, with pore sizes ranging from a few nanometers to 200 μm. This material possessed a high and stable flux rate, with ~100% cationic dye removal [68].

To overcome some of the challenges involved in the application of electrospun nanofibers for the removal of dyes, the electrospun-based nanocomposites and their blends have had to undergo development. An electrospun fiber comprising polycaprolactone/polyethylene oxide (PCL/PEO) was treated with solvent vapor annealing (SVA), followed by coating with polydopamine (PDA), to form (PCL/PEO@PDA) nanocomposite membranes. The SVA process improves the microscopic morphology of smooth (PCL/PEO) electrospun nanofibers, enhancing their specific surface area and creating active binding sites for the further depositing of PDA nanoparticles. The (PCL/PEO@PDA) nanocomposite was applied to eliminate dyes, and could be reused over several runs [69]. Additionally, nanocomposite nanofibers CA/GO were synthesized via an electrospinning approach, followed by chemical crosslinking. The surface of the fabricated nanofiber was further anchored with NH_2_-modified TiO2 nanoparticles (NPs) to form a photocatalyst composite of CA–GO/TiO_2_–NH_2_, which shows excellent photocatalytic properties for the removal of organic dyes [70].

Other types of nanocomposite nanofibers have been formed using electrospinning, with the function of removing the organic compounds and dyes from aqueous solutions. These include magnetite nanoparticles (Fe_3_O_4_)NPs modified with 3-mercaptopropionic acid, which were assembled on amidoximated polyacrilonitrile (APAN) nanofibers via an electrospinning approach followed by crosslinking [73], as well as mesoporous polyvinyl alcohol/chitosan/silica nanocomposite nanofibers (PChiCN) [77]; composite nanofibers based on polyacrylonitrile (PAN), zinc oxide (ZnO) and hinokitiol (HT) [79]; hydrophobic TiO_2_/lignin composed of a carbon nanofibers (TiO_2_@CFs) composite [81]; polyethersulfone/titanium dioxide (PES/TiO_2_) nanocomposite nanofiber membranes [84]; Ag@ZnO/TiO_2_ nanofibrous membranes with hierarchical nanostructures [85]; electrospinning reduced graphene oxide (rGO)/TiO_2_/poly(acrylonitrile-co-maleic acid (PANCMA)) nanocomposite nanofibers (Espun rGO/TiO_2_/PANCMA NFs) [86]; Cu_2_O/PLA composite nanofibers [87]; and an innovative polyvinyl alcohol (PVA)/polyacrylic acid (PAA)/MXene fiber membrane [88].

These new nanostructured materials have considerable utility as powerful adsorbents due to their good characterizations. However, some nanomaterials tend to agglomerate, and exhibit some drawbacks limiting their treatment utility. Therefore, many works have explored the use of modified electrospun nanofibrous membranes functionalized with polymer materials to overcome such limitations [61,67,73,74,75,76,77,78,83,92]. Electrospun sulfonated polyethersulfone (SPES) nanofibrous membranes are an example of such modified electrospun nanofibrous membranes; these are prepared via a two-nozzle electrospinning technique for extracting nanoparticles, dyes, and heavy metal ions from multicomponent wastewaters via the filtration and sorption of MO and other pollutants [67].

Another important example of such modified materials is the electrospun nanofiber membrane of poly(L-lactic acid) (PLLA) coated with p-toluenesulfonic acid-doped polyaniline (pTSA-PANI), which is employed for the removal of MO. The loading of PLLA membranes with pTSA-PANI improves the removal efficiency of the membranes, which can be demonstrated by the π–π interactions seen between dye-benzene rings and pTSA-PANI rings, and the excellent wettability properties of pTSA-PANI/PLLA membranes compared with PLLA membranes [74].

**Figure 2:**
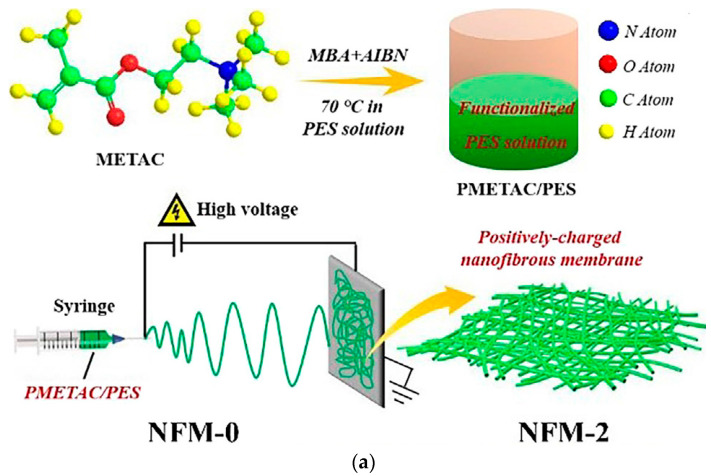

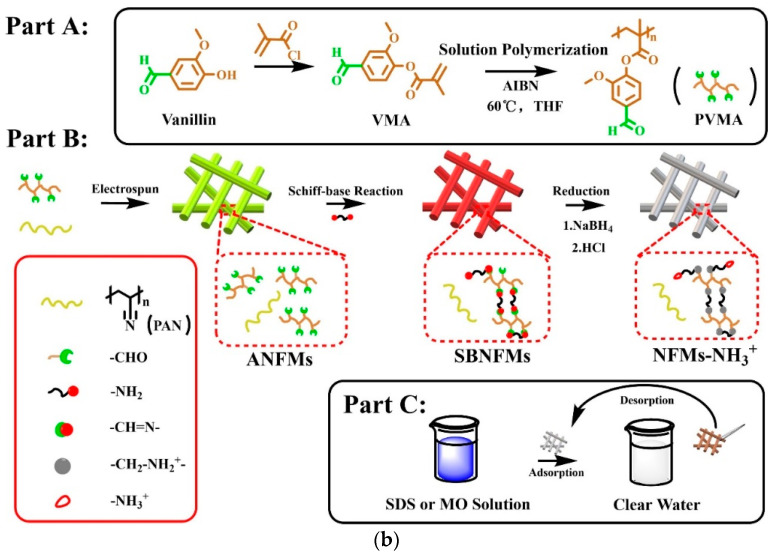
Schematic illustration of (**a**) the electrospinning of the solution and the fabrication of positively charged PES NFMs (reproduced from [75] with permission of Elsevier, 2019), and (**b**) the production of the polymer (PVMA) starting from the bio-based monomer vanillin (Part A); creating a functional membrane by sequential reactions with ethylenediamine, sodium borohydride and HCl (Part B); and the removal of SDS and MO via the membranes (Part C) (reproduced from [78] with permission of Elsevier, 2020).

Additionally, positively-charged nanofibrous membranes (NFMs) have been prepared through a combination of electrospinning and the in situ crosslinked polymerization of poly [2-(methacryloyloxy)-ethyl] trimethyl ammonium chloride (PMETAC) in poly(ether sulfone) (PES) solution. There, the quaternary ammonium salt polymer of PMETAC donated positive charges to the NFMs, enabling them to kill bacteria and adsorb anionic dyes, as represented in Figure 2a [75]. The grafting of poly(para-, ortho-, and meta-phenylenediamine (PPDA)) onto electrospun carbon nanofibers was investigated via the oxidative polymerization of o-, p-, and m-PDA onto the ECNF’s surface to form PPDA-g-ECNFs [76]. On the other hand, aldehyde-containing nanofiber membranes (ANFMs) were prepared using a vanillin biomass-derived polymer as the raw material via an electrospinning strategy.

The electrospun nanofibers with specific surface areas facilitate the accessibility of the functional aldehyde groups, and undergo an efficient Schiff base reaction after the reduction and protonation reaction. These three-step reactions transform the original hydrophobic ANFMs into a hydrophilic nanofiber mat carrying functional ammonium groups (NFMs-NH_3_^+^). The prepared membranes (NFMs-NH_3_^+^) and dodecyl sulfate contain adsorbents that help to eliminate anionic pollutants such as methyl orange, as shown in Figure 2b [78]. Via the electrospinning strategy, many membranes, such as a polyetherimide (PEI) nanofiber functionalized by anchoring with titanium oxide (TiO_2_) [83] and polyamide/polyethyleneimine (PA/PEI) nanofibers [92], were fabricated and utilized as promising nanomaterials for wastewater treatment applications. Recently, the roles of metallic ion nanoparticles in the photocatalytic degradation of organic dyes and other organic pollutants using solar energy have attracted great attention as an effective way to solve critical environmental issues.

However, these metallic ion nanoparticle catalysts are generally found in powder forms, which easily aggregate in liquid media and form a suspension that cannot be recovered or separated after use [80]. We have investigated a way to avoid such problems, and the catalytic activities of the synthetic nanofibers were examined in relation to some organic dyes. Zirconia or zirconium oxide (ZrO_2_) fibers were fabricated using sol-gel and electrospinning strategies, utilizing polyvinylpyrrolidone and zirconium butoxide as precursors, followed by treatments at various temperatures and examination for dye removal [72].

Additionally, a novel flexible TiO_2_/N-doped carbon nanofiber (TiO_2_/NCNF) mat with “memory catalysis” activity was prepared by combining electrospinning and carbonization methods. In contrast to the structure of TiO_2_/NCNFs, the addition of a Ti resource to the PAN solution leads to the self-assembly of TiO_2_ in the NCNF support, enhancing the flexibility and mechanical functionality of the catalysts, providing highly active sites, and ensuring easy recovery from treated water. Moreover, the N-graphitized carbon can collect the electrons released from TiO_2_ under irradiation light, and then reuse them in the dark to perform “memory catalysis” [80]. In addition to TiO_2_, zinc oxide (ZnO) is found to be one of the most commonly applied metal oxides due to its promising intrinsic properties; it has greater photosensitivity and wide bandgaps, and has a porous structure [82]. As such, ZnO nanofibers were manufactured via the electrospinning of the PVA/ZnAc solution, followed by calcination at various temperatures. Subsequently, the calcined samples were subjected to gradual or rapid cooling to obtain highly photocatalytically active ZnO nanofibers. The findings reveal that this rapid cooling approach enhances the crystallinity, surface area and porosity of the ZnO nanofibers, as well as the binding sites contained within [82].

Additionally, catalytically active porous and hollow titania nanofibers encapsulating gold nanoparticles (AuNPs) were prepared using the conjugation of sol-gel chemistry and a coaxial electrospinning strategy. This strategy involves a shell composed of a mixture of PVP and titania sol, while the core is composed of a mixture of poly(4-vinyl pyridine) (P4VP) and pre-prepared AuNPs. The core–shell nanofibers were thermally treated stepwise up to 600 °C, which caused the dissociation and decomposition of the organic materials of the nanofibers. This behavior gave rise to porous and hollow titania nanofibers, with the catalytic AuNPs established in the inner wall of the titania shell [89]. On the other hand, cobalt nanoparticles (Co-NPs) are well known as a metal-based catalyst with high catalytic performance, but their aggregation during catalytic operations limits their catalytic activities, and gives rise to difficulties in their recovery after reactions [90]. Thus, cobalt-loaded activated carbon nanofibers (Co/C)NFs were synthesized by one-pot electrospinning after thermal treatment, and we tested their performance in the catalytic degradation of sulfamethoxazole (SMX) [90].

To prevent the agglomeration of bimetallic composites, the SMX was immobilized on support materials, and a polymeric membrane for surface impregnation was utilized in the treatment of chlorinated organic compounds [91]. Recent work has investigated the fabrication of bimetallic Fe/Ag nanoparticles loaded on electrospun polyacrylonitrile nanofibers (PANNFs) pre-modified with EDTA and ethylenediamine (EDA) as the chelating agents. The catalytic activities of the fabricated nanofiber mat were studied in the context of degrading azo-dyes [91].

### 5.2. Removal of Metals

In the last decade, many researchers have focused their work on removing the inorganic pollutants from wastewater using nanofibers fabricated via the electrospinning technique, as shown in Table 2 [7,8,9,93,94,95,96,97,98,99,100,101,102,103,104,105,106,107,108,109,110,111,112,113,114,115,116,117,118,119,120,121,122,123,124,125,126,127,128,129,130,131,132,133,134,135,136,137,138,139]. As such, multifunctional electrospun nanofibers have been fabricated and widely utilized in the last few years to remove various types of inorganic pollutants, such as lanthanides and actinides [7,93,94], Pb^2+^ (with other metals ions) [95,96,97,98,99,100,101,102,110,111,112,113,114,115,118,119,121], Cu^2+^ [8,9,102,103,104,105,117,120], Cd^2+^ [106,107,108], Ni^2+^ [109], Cd^2+^, Zn^2+^ [116], Ca^2+^, Mg^2+^ [122], heavy metals [123,124], Cr^6+^ [125,126,127,128,129,130,131,134,135,136,137,138,139], and As^5+^ [132,133].

#### 5.2.1. Removal of Some Lanthanide and Actinide

For lanthanide and actinide, a poly(vinyl alcohol)/sodium hexametaphosphate hydrogel nanofiber (PVA/SHMP HENF) with a 3D structure was prepared via an electrospinning technique in one step. The crosslinking reaction was carried out by thermal treatment using citric acid (CA) as the crosslinking agent. The fabricated PVA/SHMP HENF showed high swelling properties, high pH stability, and the remarkable removal for some lanthanide metals, such as La^3+^, Tb^3+^, and Nd^3+^. Moreover, it can be regenerated and reused for about eight cycles [93]. Additionally, PAN-272 nanofiber membranes were fabricated via the integration of PAN electrospun nanofiber with a commercial organic extractant, Cyanex272, to enhance its efficiency against lanthanide ions, such as Eu^3+^ and Y^3+^ [7]. The obtained data show that the fabricated nanofiber membrane is a promising material for use in recovering lanthanide metal ions. On the other hand, polymer (polyacrylonitrile; PAN) nanofibers were chemically modified for application with lower-pressure-reactive filtration to adsorb uranium (VI) (U^6+^). The binding reagents were either nitrogen-based (Aliquat^®^336(Aq)) or phosphorous-containing hexadecylphosphonic acid (HPDA). Then, bis(2-ethylhexyl) phosphate (HDEHP) was mixed (at 1–3 wt. %) with PAN sol-gel, and we utilized electrospinning to yield a modified nanofibers membrane. For comparison, PAN nanofibers were also loaded with amidoxime (AO) moieties, a group with a high capacity to adsorb uranium (VI) (U^6+^). Aq-containing materials containing quaternary ammonium groups appear to sequester U^6+^ by electrostatic interactions; the adsorption rates of such materials are limited, and will be high at pH = 7, where positively charged N-groups attract negatively charged complexes of uranium. On the other hand, HDPA and HDEHP act effectively at acidic pH levels, where the surface complexation of U^6+^ likely drives absorptivity. Complexation using amidoxime was effective at different pH values, although U^6+^ removal through surface precipitation may also occur at circumneutral pH values and with higher dissolved uranium concentrations [94].

#### 5.2.2. Removal of Heavy Metals

Electrospun nanofibers play vital roles in reducing and taking heavy metals from wastewater; heavy metals are considered a serious environmental pollutant, and pose a tremendous threat to human health and other living things. Heavy metal ions such as Cd, Cu, Pb, and Hg are regarded as a major class of inorganic contaminants that pollute water and have dangerous physiological impacts [54], along with posing major threats to water resources [6]. Additionally, they are difficult to decompose, and accumulate in the bodies of organisms. In many cases, the levels of these metal ions in natural drinking water are beyond allowable limits. In the last few years, different nanofibrous nanomaterials fabricated by electrospinning techniques have been recognized as extremely efficient materials that can be utilized to remove heavy metal ions. The α-Fe_2_O_3_/PAN nanofibers are an example of these promising nanofibers, which are fabricated by the electrospinning technique followed by a simple hydrothermal process [95]. In this process, a polyacrylonitrile (PAN) nanofiber mesh is anchored with α-Fe_2_O_3_ (α-Fe_2_O_3_/PAN) as an effective adsorbent, and this is used to remove Pb^2+^ from wastewater [95].

**Figure 3:**
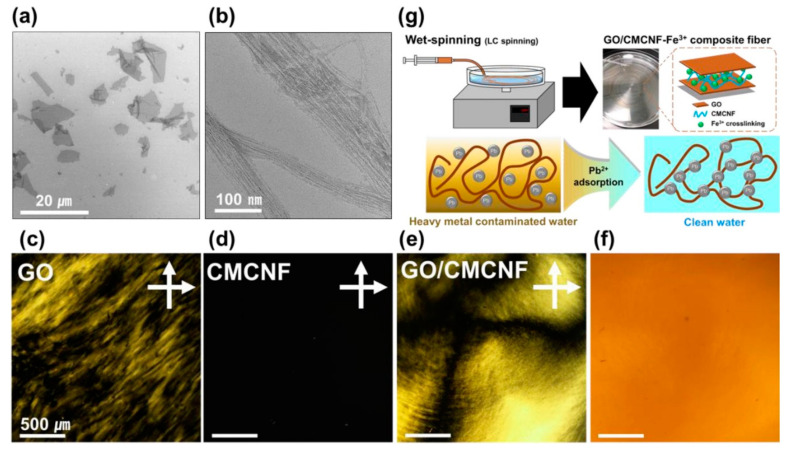
(**a**) SEM images of exfoliated GO sheets. (**b**) TEM image of CMCNF morphology. POM images of (**c**) GO at 0.5 wt. %, (**d**) CMCNF at 2 wt. %, (**e**) GO/CMCNF (5/5) mixture at 0.5 wt. %. (**f**) Optical microscope image of an identical area corresponding to (**e**). (**g**) Schematic diagram of GO/CMCNF-Fe^3+^ CF with Pb^2+^ adsorption from aqueous media (reproduced from [96] with permission of Elsevier, 2020).

Furthermore, GO has gained great attention in the decontamination of heavy metal ions from aqueous media, but it has many disadvantages, as it can be dispersed in a liquid phase and cannot be recovered easily. Consequently, a new composite fiber (CF), made of a GO/carboxymethyl cellulose nanofibril (CMCNF) composite, was fabricated as an effective and durable adsorbent for Pb^2+^, as illustrated in Figure 3 [96]. GO/CMCNF CF was crosslinked using an Fe^3+^ ion as a coagulant during a typical wet-spinning process. Based on various interactions, such as ionic bonding and electrostatic interactions between Fe^3+^ and the -COOH groups on CMCNF, the CF presented a better mechanical function than the pure GO fiber. The GO/CMCNF-Fe^3+^ CF enabled enhanced lead (Pb^2+^) adsorption [96]. Additionally, a polycaprolactone nanofibrous material was made by the electrospinning approach, followed by functionalization with clay minerals and zeolite nanoparticles, and this was applied in the removal of Pb^2+^ from an aqueous solution [97]. The removal of Pb^2+^, along with other metal cations such as Cu^2+^, Ca^2+^, Co^2+^, Zn^2+^, and Ni^2+^, was investigated using a macrocyclic pyridone pentamer-modified nanofiber prepared via electrospinning [98]. The regeneration of the fabricated nanofibers was carried out successfully using an ethylenediaminetetraacetic acid solution [98].

**Figure 4:**
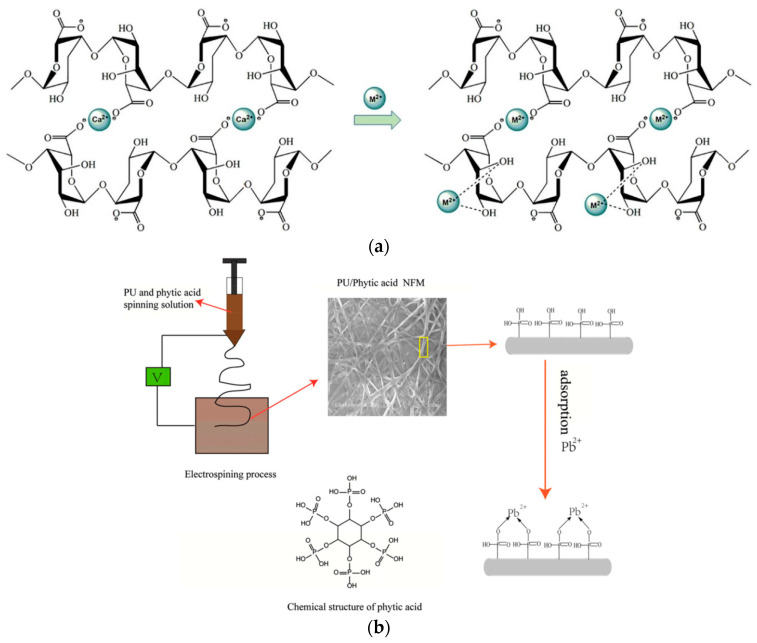
(**a**) Possible binding mechanism of alginate composite fibers with heavy metal ions. M^2+^ represents the Pb^2+^ or Cu^2+^ ion (reproduced from [99] with permission of Elsevier, 2019), and (**b**) the chemical structure of phytic acid, the electrospinning process, and the absorption mechanism of Pb^2+^ (reproduced from [100] with permission of Taylor and Francis, 2019).

To overcome the disadvantages of the application of calcium alginate aerogels to adsorb heavy metals, a novel alginate composite nanofiber with enhanced toughness was fabricated by electrospinning, using GO as the reinforcing filler, for adsorbing Pb^2+^ ions from aqueous media. It achieved considerable adsorptive capacity due to the synthesized round-shaped nanofibers, as shown in Figure 4a [99]. Electrospinning has also been utilized to fabricate polyurethane (PU) PU/phytic acid nanofibrous membranes to remove Pb^2+^ from aqueous media. The addition of phytic acid enhances the hydrophilicity and decreases the mechanical features to a certain extent, but protects certain mechanical properties, as represented [100].

Recent publications have reported various types of novel electrospun nanofibers with promising properties, used for the removal of Pb^2+^ from aqueous media, such as electrospun nanofibers coated with a hydrogel layer with a tunable thickness [101], and electrospun superamphiphilic (SiO_2_/TiO_2_) porous nanofibers membranes (STPNMs) composed of interfiber mesopores and interfiber macropores, and functionalized by an amino-salinization reaction to yield the membrane (ASTPNMs) [102]. Additionally, several electrospinning nanofibers were fabricated to adsorb copper (Cu^2+^) and (Pb^2+^), such as crosslink polyvinyl alcohol (PVA) nanofibers [110], mesoporous PAA/dextran-polyaniline core–shell nanofibers [111], and nanocomposite membranes (MWCNT-PEI/PAN) formed from multiwalled carbon nanotubes (MWCNTs) with polyethyleneimine (PEI) mixed with polyacrylonitrile (PAN) nanofibers [112].

In the presence of other heavy metal ions such as cadmium (Cd^2+^), many electrospinning nanofibers were synthesized with improved absorptivity and selectivity. A poly(vinyl alcohol)/chitosan (PVA/Chi) nanofiber membrane was created by the electrospinning method for the selective and improved uptake of lead (Pb^2+^) and cadmium (Cd^2+^) ions from acidic solutions [113]. The fabricated nanofiber membranes show high selectivity towards Pb^2+^ and Cd^2+^ ions in the presence of competing ions [133]. Additionally, a (PAN)/polyaniline (PANI)-nylon core–shell nanofiber membrane was fabricated as a filtration–adsorption membrane, and its surface was functionalized with diethylenetriamine (DETA) for the sorption of Pb^2+^ and Cd^2+^ ions from aqueous solutions. The functionalization process improves the hydrophilicity properties of the membrane, giving rise to better antifouling features and higher permeability [114]. PAN/chitosan/UiO-66-NH_2_ composite nanofibers were fabricated using the electrospinning technique to eliminate Pb^2+^, Cd^2+^, and Cr^6+^ ions—via the adsorption and membrane filtration methods—from aqueous solutions [115]. Likewise, a novel adsorbent 2,3,6 Tricarboxy cellulose nanofiber (TPC-CNFs) was fabricated by the 2,2,6,6-tetramethylpiperidine-1-oxyl (TEMPO) oxidation of dissolved cellulose pulp (selective at C-6), followed by periodate-chlorite oxidation (selective on C-2 and C-3). A tricarboxy cellulose nanofiber was investigated as a novel sorbent for Ca^2+^, Pb^2+^, and Cu^2+^ in aqueous solutions [119].

For copper ion (Cu^2+^), the excessive accumulation of heavy metal ions, such as (Cu^2+^), in the surrounding environment could cause many diseases and will pose detrimental effects. Therefore, the removal of these metals ions and their harmful effects has attracted the interest of numerous researchers. Electrospinning nanofibers offers an effective and sustainable way of producing a suitable nanomaterial for the removal of these metal ions. Porous poly(L-lactic acid) (PLLA) nanofiber membranes with a large surface area were created by electrospinning and post-acetone curing, and acted as a substrate to deposit chitosan (Chi) and remove Cu^2+^ from aqueous solutions. Chi was covered onto a porous nanofibrous membrane through a direct soaking coating method, and acted as an efficient sorbent due to –NH_2_ and –OH, which are chelating sites for the chelation of Cu^2+^ [17]. Additionally, a novel nanofibrous polyethersulfone-poly(dimethylamino) ethyl methacrylate was fabricated to remove Cu^2+^ from aqueous solutions [103].

On the other hand, different types of bio-polymer, such as proteins, can be considered promising materials for absorbing heavy metal ions via their functional groups. Barley is one of the most important protein sources in the world, and it contains about 80.13% protein. Consequently, Guan et al. (2019) fabricated hordein nanofibers via the electrospinning method [104]. This work fabricated hordein and hordein/N,N′-methylene bisacrylamide (MBA) nanofiber membranes. The synthesized membranes were loaded with β-cyclodextrin (β-CD) to eliminate heavy metal ions from aqueous media. It was noted that the introduction of MBA and β-CD at optimal concentrations could significantly enhance the adsorption capacities of Cu^2+^ [104]. Additionally, via the electrospinning method, a type of tree-like porous carboxyl functionalized cellulose nanofiber membrane was constructed to remove Cu^2+^ [18]. The presence of TBAC caused the formation of tree-like structures, and MnO_2_ nanoparticles were applied as pore-forming reagents. Carboxyl groups (–COOH) ionized into carboxylate ions (–COO^-^) have a high affinity for Cu^2+^ uptake. Moreover, the tree-like porous structure provided a high specific surface area, and enhanced the adsorption capacities [18]. Additionally, poly(acrylic acid)-sodium alginate nanofibrous hydrogels (PAA-SA-NFHs) were prepared via electrospinning, and crosslinked using thermal treatment. The PAA-SA-NFHs, with a high swelling percent, intrinsic fiber morphology, good stability, and suitable mechanical properties, presented high adsorption affinities towards Cu^2+^ [105].

Furthermore, this work has outlined a sustainable approach to transforming toxic Cu^2+^ pollutants into valuable Cu nanoparticles that can increase the functionality of catalysis, and the fabricated PAA-SA-NFHs with uniformly coated Cu-nanoparticles showed a preformed catalytic activity [105]. Other electrospinning nanofibers were fabricated via the electrospinning approach to remove Cu^2+^ and other metals ions, such as (TiO_2_/ZnO) nanofibers, which anchor onto the sewage sludge carbon’s (SSC) surface for the removal of Cu^2+^, Ni^2+^, and COD from aqueous solutions [117]. Furthermore, phosphorylated polyacrylonitrile-based (P-PAN) nanofibers were fabricated via the electrospinning method, and applied for the adsorbing of Cu^2+^, Ni^2+^, Cd^2+^, and Ag^+^ from an aqueous solution [120].

As regards Cd^2+^, in the processing and refining of many raw ore minerals such as zinc sources, cadmium occurs naturally, and is mainly leached during the refining process as a by-product. Cd has very dangerous effects on human health and ecosystems, where it bioaccumulates in plants, invertebrates, and vertebrates. In recent works, electrospun nanofibers have been investigated as suitable nanomaterials to remove Cd^2+^. A nanofibrous electrospun nonwoven adsorbent, prepared from chitosan and phosphorylated nanocellulose (PNC), was investigated for use in the uptake of Cd^2+^ ions from aqueous media, for the sake of water sustainability [107]. A composite nanofiber based on poly(vinyl alcohol) (PVA) and SA was fabricated to adsorb Cd^2+^ ions from aqueous media [108]. To achieve this objective, a polymer solution composed of PVA (10 wt. %)/SA (2 wt. %) at three-volume ratios of 0/10, 1/4, and 2/3 was first investigated, and after that, an electrospinning process was used to prepare the nanofibers [108]. Other nanofibrous mats were fabricated and investigated to remove Cd^2+^ associated with other metals ions [116,118,121]. Highly efficient and affordable composite nanofibrous mats were fabricated to remove Cd^2+^ and Zn^2+^ from an aqueous solution [116]. These composite nanofibrous mats were prepared from cellulose acetate (CA) and rectorite (REC), which were then loaded with biosorbent saccharomyces cerevisiae (SCV) to produce a sandwich-like structure. The -OH and C=O groups in the CA donated nanofibrous mats with a high affinity for adsorbing heavy metals. The REC, which was incorporated into CA to make a CA/REC nanofibrous membrane, enhanced the nanofibers’ specific surface area, and presented ideal scaffolds for conjugation with SCV, enabling the enhancement of the affinity among the nanofibrous mats and heavy metal ions [116]. A developed electrospun (CNFs/TiO_2_-PAN) hybrid membrane was fabricated and examined for use in the extraction of various metal ions (such as Cd^2+^, Pb^2+^, Cu^2+^) and cationic dyes (such as methylene blue (MB)) from polluted drinking water sources [118]. Highly effective membranes based on electrospun polycyclodextrin (poly-CD) nanofibers were synthesized via the electrospinning of CD molecules in the presence of a crosslinker (i.e., 1,2,3,4-butane tetracarboxylic acid), and tested for the elimination of numerous PAH compounds (i.e., acenaphthene, fluorene, fluoranthene, phenanthrene, and pyrene) and heavy metals (i.e., Cd^2+^, Pb^2+^, Ni^2+^, Mn^2+^, Zn^2+^, and Cu^2+^) from wastewater over time [121].

Recently, the electrospinning method was applied to fabricate various types of nanofibers, such as cellulose acetate (CA) nanofiber with various contents (0, 1.0, 1.2, and 1.4 wt. %) of iron-modified nanozeolite (Fe-MNZ). The prepared nanofibers removed Ni2+ ions from simulated wastewater [109]. Furthermore, cellulose acetate nanofibers (CANFs) were prepared and then deacetylated to form CNFs anionized via a pad-batch method. Anionic–cellulose nanofibers (a-CNFs) with a carboxymethyl group were tested for use as adsorbents of Ca^2+^ and Mg^2+^ [122]. Additionally, a β-cyclodextrin (β-CD)/Chi/PVA nanofiber membrane was fabricated to remove organic and heavy metal micropollutants from drinking and wastewaters [124].

The contamination of water resources with toxic Cr^6+^ ions is a pressing issue for the environment and human health, given its high toxicity and carcinogenicity. It is discharged into the environment along with other metal ions from the mineral, textile, paint, and petroleum refining industries. During the last few years, electrospun nanofiber mats have been created that function via numerous wastewater purification techniques. Therefore, the unique characteristics of electrospun nanofibrous membranes have attracted great attention. Accordingly, many electrospun nanofibrous membranes have been fabricated and utilized to remove Cr^6+^ from aqueous media [125,126,127,128,129,130,131,134,135,136,137,138,139]. A series of poly(acrylonitrile-butadiene-styrene)/polyacrylonitrile–ZnO (ABS/PAN–ZnO) membranes was prepared by coaxial electrospinning. The coaxial electrospinning of ABS and PAN solutions with various ZnO nanoparticle contents was employed to remove Cr^6+^ ions via the photoreduction strategy in water purification processes [125]. Additionally, zerovalent iron (ZVI) and cerium oxide nanoparticles, (CeO_2_)NPs, doped into PAN nanofibers were applied for the removal of Cr^6+^ from an aqueous solution [126]. Other new nanofibrous materials have been synthesized in the last few years via electrospinning strategies to remove Cr^6+^, such as free-standing PVA/PAN nanofibrous composite membranes with interpenetrating nanofibers structures [127], the PAN nanofiber mat (n-fib@Mat), the PAN/ZnO n-fib@Mat, the PAN/ZnO-TiO_2_ n-fib@Mat [128], environment-friendly biosorbent cellulose nanofibrous mats [129], novel composite nanofibers based on PAN/GO [130], and uniform chitosan composite (Chi/PAAS) nanofibrous materials loaded with 4.0 wt. % polyacrylic acid sodium [131].

Other toxic heavy metal ions, including selenium (Se^4+^), arsenic (As^5+^), cadmium (Cd^2+^), iron (Fe), copper (Cu^2+^), and cobalt (Co^2+^), can co-exist with Cr^6+^ ions; these metal ions have higher toxic effects compared with the same metal ions in another oxidation state. For example, Se^4+^ exhibits about 10 times higher toxicity than Se^6+^ [134]. The separation of such toxic ions has gained considerable attention from scientists worldwide [134,135,136,137,138,139]. Se^4+^ and Cr^6+^ ions were eliminated from aqueous solutions by magnetite nanoparticles (MNPs)/graphene (G) nanosheets doped with polyvinyl alcohol (PVA) and cellulose acetate (CA) electrospun nanofibers [134]. Additionally, electrospun nanofibers made of cellulose acetate/chitosan/single-walled carbon nanotubes/ferrite/titanium dioxide (CA/Chi/SWCNT/Fe_3_O_4_/TiO_2_) were fabricated and studied for the adsorption of Cr^6+^ and As^5+^ [135]. On the other hand, via the electrospinning technique, chitosan-integrated poly(N-vinyl caprolactam) (Chi-g-PNVCL) nanofibers were developed, and ZIF-8 metal–organic framework nanoparticles were anchored into the nanofibers to remove Cr^6+^ and As^5+^ from water [136]. The synthesized nanofibers could be reused in five sorption–desorption runs [136].

In the last decade, electrospun nanofiber membranes have been widely investigated and utilized for wastewater treatment. One of the most effective membranes are nanofibrous composite membranes based on polyvinyl alcohol (PVA) and polyacrylonitrile (PAN) [137], which are synthesized via the two-nozzle electrospinning strategy and utilized to remove both nanoparticles and heavy metal ions, such as Cr^6+^ and Cd^2+^, from wastewater [137]. To develop an organic membrane with many mechanical properties and to overcome the main challenges facing the fabrication of electrospun agave cellulose, composite membranes composed of polycaprolactone (PCL) and cellulose nanofibers (CNF) in various compositions were synthesized via the electrospinning strategy, and investigated for use in the removal of Cr^6+^ and Fe contaminants from tap water, where the cellulose was extracted from agave bagasse [138]. To improve the adsorption capability of chitosan in decontamination processes, Chi, poly(glycidyl methacrylate) (PGMA), and polyethyleneimine (PEI) were sequentially integrated onto the Chi electrospun membrane’s surface to enrich amino-modified Chi electrospun membranes (Chi-PGMA-PEI) for the removal of heavy metal ions (Cr^6+^, Cu^2+^, and Co^2+^) [139].

On the other hand, various processes have been investigated for the removal and elimination of arsenate from arsenate-containing water. Many electrospun nanofibers have been identified as novel adsorbents of great significance [132,133]. Lanthanum loaded on an electrospun chitosan nanofiber (ChiN-La) is an example. The attractive behavior of ChiN-La is rooted in its unique structural properties [132]. The host material Chi-NFs can preconcentrate the arsenate via electrostatic attractions, and the loaded lanthanum enables arsenate adsorption via specific interactions [132]. Additionally, electrospun nanofiber membranes of PAN, loaded via the dispersion of α-Fe_2_O_3_ nanoparticles that were prepared via a thermal solvent process, in a polyacrylonitrile solution were investigated for the removal of As^5+^. The nanofiber achieved the excellent removal of As^5+^ using batch and filtration techniques [133].

### 5.3. Oil/Water (O/W) Separation

Annually, large volumes of oily sewage are discharged into the environment due to oil spill incidents, and other oil-related activities, such as those of the food, textile, tannery, and metallurgy industries, which have caused serious water pollution problems [46]. Consequently, scientists direct their attention towards the treatment and separation of oil–water mixtures with different technologies to solve these problems [140]. Various nanomaterials, nanofibers, and techniques have been developed to decontaminate and separate oil–water mixtures [141]. Due to the unique characteristics and advantages of electrospun nanofibrous materials, such as their higher porosity with a high internal area, low density, reusability, and controllable pore size, they play a vital role, and have gain great interest as oil absorbents used to collect oil spills from water surfaces [141,142,143,144,145]. Many published works have focused on developing and applying nanofibrous membranes and multifunctional electrospun nanofibers to purify oily wastewater, as represented in Table 3 [141,142,143,144,145,146,147,148,149,150,151,152,153,154]. The data illustrate that nanofiber membranes are used as effective oil sorbents on a large scale to remove oil spills from the water’s surface, with promising results.

Membrane technology has been successfully utilized to treat and purify different wastewaters due to its considerable economic and technical merits [140]. However, the traditional nanomaterials used as membranes have some limitations, such as being non-degradable, leading to further disposal issues after usage [141]. Consequently, many works investigate using environmentally friendly materials, which have properties of degradability, renewability, non-toxicity, and good mechanical properties [140,141,142]. Polylactide (PLA) is considered one of the most promising eco-friendly polymers manufactured as a biodegradable separation material [140,141,142]. Therefore, polylactic acid-based nanofiber membranes loaded with metal oxides (SiO_2_, TiO_2_, Al_2_O_3_, and CeO_2_) were prepared via the blow-spinning of a blended solution of polylactic acid (PLA) and metal oxide nanoparticles (NPs). The obtained results show that introducing SiO_2_ NPs massively increases the hydrophobicity of the membranes. The PLA/SiO_2_ nanofiber membranes possessed a higher separation efficiency than pure PLA, PLA/TiO_2_, PLA/Al_2_O_3_, and PLA/CeO_2_ nanofiber membranes, with good separation performance (~100%) and permeation flux (17,800 L m^−2^ h^−1^ for n-heptane), as well as a prominent oil sorption capacity (19.9 g/g for n-hexane) [140]. Additionally, superhydrophobic polylactide (PLA) nanofibers were prepared via an electrospinning method, and reacted with a hydrophobic agent, alkyl ketene dimer (AKD). Alkyl ketene dimer comprises functional groups that react specifically with –OH groups. Glycerol is used as a template, introducing –OH functional groups into the PLA matrix. The electrospun nanofiber membranes were then placed in (AKD) solutions, in which an integration reaction occurred, resulting in the improved hydrophobicity of the fiber filter. The fabricated nanofiber material showed excellent oil removal rates, with sorption capacities reaching 10 gg^−1^ and the ability to run for over 10 adsorption–desorption cycles [141]. On the other hand, biodegradable hybrid nanofibers membranes of PLA and ZIF-8@C600 were fabricated using electrospinning. The fabricated (PLA/ZIF-8@C600) porous nanofiber mat have excellent oil absorption capacities in oil/water mixtures and emulsion. The oil sorption capacity of a sample with a 1 wt. % content of ZIF-8@C600 was improved by ~72.59% when using pure poly(lactic acid) [142].

**Figure 5:**
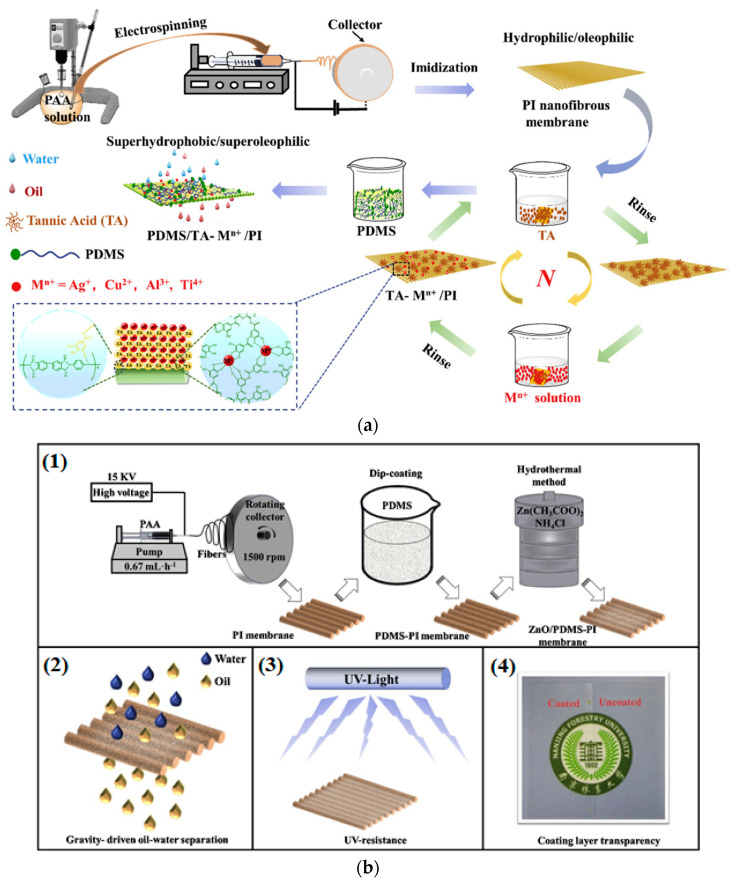
Schematic illustration for the fabrication of (**a**) PDMS/TA-Mn+/PI nanofibrous membrane and the formation mechanism of the PDMS/TA-Mn^+^ coating on the PI nanofibrous membrane (reproduced from [143] with permission of Elsevier, 2019), and (**b**) the ZnO/PDMS-PI membrane. (**1**) The preparation process, (**2**) the scheme of gravity-driven oily wastewater separation, (**3**) the UV-irradiation experiment, and (**4**) the transparency of the coating layer (reproduced from [144] with permission of Elsevier, 2019).

Recently, metal–phenolic coordination materials have gained great attention in the engineering of functional nanomaterials when seeking to fabricate multifunctional nanofibrous membranes that perform well in different oil/water mixtures [143]. Consequently, a multifunctional superhydrophobic nanofibrous membrane was prepared by applying a simple and novel nature-inspired approach, whereby a polyphenol (tannic acid) metal complex is added to form a rugged hierarchy on the surface of an electrospun polyimide (PI) nanofiber membrane, followed by the functionalization of poly(dimethylsiloxane) (PDM), as illustrated in Figure 5a. The results show that the as-synthesized tannic acid−Al^3+^-based superhydrophobic membrane presents anti-impact, low-adhesive and self-cleaning properties, and furthermore, has a high capacity to purify oil/water mixtures: a high flux up to 6935 Lm^−2^ h^−1^, with a separation ability of >99% in water containing oil at <5 ppm, even after 20 uses. In addition, the membrane shows considerable UV-shielding function due to the inherent UV-absorbing property of tannic acid.

Moreover, the membrane also has an antibacterial ability, high biocompatibility, high mechanical strength, and good resistance to harsh conditions [143]. Additionally, a UV-resist and transparent coating based on PDMS and ZnO anchored on a highly stable and self-standing polyimide was investigated and is represented in Figure 5b. The resulting fibrous membranes possessed superior UV-resistant abilities and high oily wastewater separation efficiency (higher than 99%). Moreover, the composite membrane showed superoleophilicity and hydrophobicity properties in harsh conditions, and the transparent anchoring layer can be used in other applications, such as UV-blocking [144].

Some raw materials such as polyvinylidene fluoride (PVDF) are considered amongst the most common utilized in the fabrication of materials used in separation techniques. PVDF has promising properties, such as perfect chemical/thermal stability, high mechanical properties, as well as excellent film/fiber fabrication characteristics [145]. Therefore, it is used to manufacture PVDF/rGO/TiO_2_ nanofiber webs for oil/water separation by adding hydrothermally prepared rGO/TiO_2_ nanoparticles into an electrospun solution. From oil–water separation studies, it can be noted that PVDF/rGO/TiO_2_ nanofiber membranes with an rGO/TiO_2_ quantity of 3% have the best oil removal abilities, at 98.46% [145].

**Figure 6:**
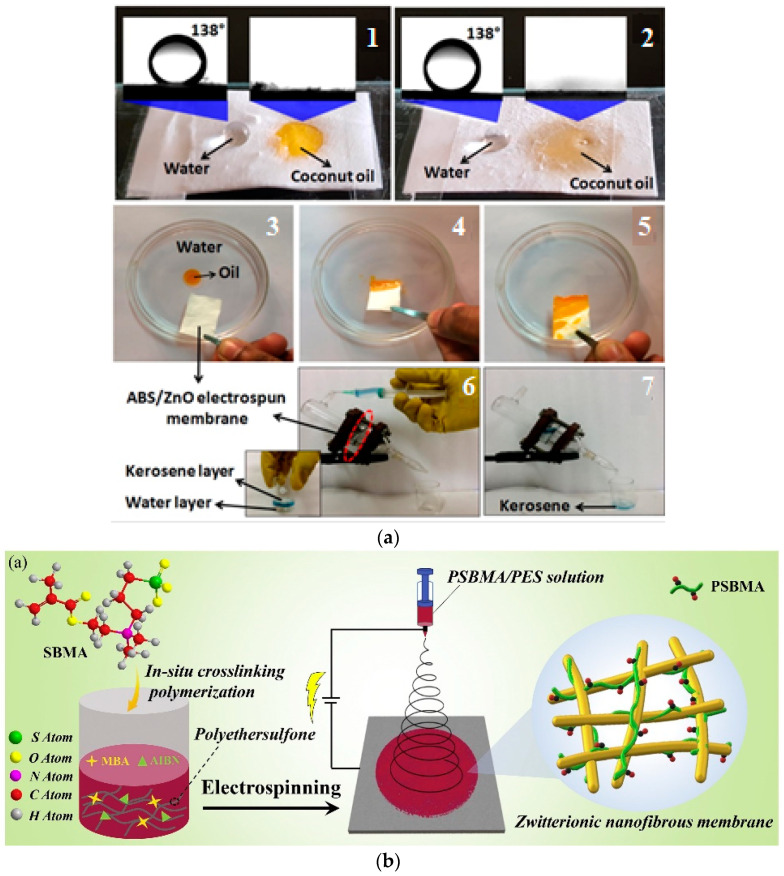

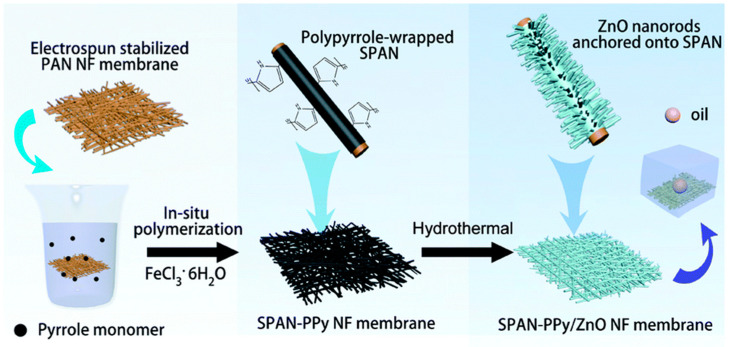
(**a**) Photographs of water and oil absorption characteristics of an electrospun membrane (**1**) ABS, (**2**) ABS/ZnO; (**3**–**5**) selective absorption process of ABS/ZnO samples from the oil–water mixture; and (**6**–**7**) gravity-driven oil–water separation (reproduced from [146] with permission of Elsevier, 2019). (**b**) Schematic illustration of the fabrication of PSBMA/PES NFMs (reproduced from [148] with permission of Elsevier, 2020). (**c**) Schematic illustration of the fabrication of a SPAN-PPy/ZnO nanofibrous membrane (reproduced from [151] with the permission of the Royal Society of Chemistry, 2020).

The surfaces wettability of some polymeric membranes can be enhanced by the addition of certain metal oxides nanoparticles, such as (ZnO-NPs), which can contribute to improving the antibacterial property of ZnO-NP functionalized electrospun polyacrylonitrile membranes [146]. Hence, a superoleophobic and antibacterial nanofiber poly(acrylonitrile-co-butadiene-co-styrene) [ABS]/ZnO-NPs electrospun nanofiber nanocomposite membrane was investigated. The electrospun ABS membrane was anchored with floral zinc oxide nanoparticles using a post-treatment approach, as illustrated in Figure 6a. The pure ABS and nanocomposite membranes presented superoleophobic behaviors, and could selectively remove various oils from oil/water mixtures under gravity. The ZnO-NPs in the nanofiber could improve the oil flux, and impart an antibacterial ability onto electrospun ABS membranes againt Escherichia coli and Staphylococcus aureus [146]. Additionally, PAN nanofibers were fabricated by electrospinning, and then they were immobilized in a heterostructured PPy/ZnO layer with a hierarchical design layered onto the surface of the nanofiber, as shown in Figure 6b. The SPAN–PPy/ZnO nanofiber membrane showed super hydrophilicity and underwater superoleophobicity (>150) against various oils, excellent mechanical strength, and good underwater oil intrusion pressure (>120 kPa). Interestingly, the as-prepared nanofiber membrane presented ultra-rapid permeation flux and a good differentiation performance for oil/dye/water emulsions, but it also exhibited strong reversion abilities (96%) from organic decontamination after organic decontamination via 20 min exposure to light irradiation [141].

In the last few years, to improve antifouling membranes, three main types of hydrophilic additives, known as inorganic NPs, and hydrophilic and amphiphilic polymers, have been utilized to adjust the surfaces of fabricated membranes [148]. Zwitterionic polymers are recognized as a key additive to prevent nonspecified fouling from modifying membranes [146]. Consequently, high-stability and preformed zwitterionic nanofiber membranes (NFMs) with self-cleaning capacities were fabricated through the conjugation and in situ crosslinking of poly-(sulfobetaine-methacrylate) (PSBMA) and electrospun poly-(ether-sulfone) (PES) nanofibers, as illustrated in Figure 6c. Due to the presence of zwitterionic modified groups, the PSBMA/PES NFM showed superior antifouling activity, and exhibited significant chemical stability in acidic, alkaline, and salty media, along with a good separation ability in both layered oil/water mixtures as well as oil-in-water emulsions. Additionally, the membrane can reject bacteria and continuously separate oil/water mixtures [148].

Carbon nanotubes (CNTs) recently gained much attention due to their promising properties. Therefore, the electrospinning technique was used to produce thin-film nanofibrous nanocomposite (TFNC) membranes with notable antifouling and self-cleaning characteristics [149]. As such, an ultrathin CNTs–PVA hybrid coating layer introduced a novel super wetting membrane onto an electrospun nanofiber substrate with a highly porous structure and good mechanical properties. The 3D CNT composite coating layers were loaded onto the surface of the nanofiber substrate by crosslinking the carbon nanotubes with PVA. These covering layers act as a functional barrier to separate oil droplets, which thus show high suitability for use in treating the surfactant-stabilized oil-in-water emulsions, and giving them a higher separation ratio and considerable competitive flux under low pressure [149].

The fabrication of a mesh filter membrane to separate water/oil with higher permeating flux and high oil intrusion pressure has remained a significant and considerable challenge [153].

The ultrathin microporous membrane was investigated by coating a stainless steel mesh filter with electrospun PAA-embedded poly(vinylidene fluoride) to form PAA-g-PVDF nanofibers. The network form of the nanofiber layer decreased the size of the pore of the stainless steel mesh and improved its oil pressure rejection capacity, while keeping a highly efficient separation area to maintain the extreme permeability function of the mesh filter membrane, along with the high permeating flux and high oil intrusion pressure at the same time [153]. The fabrication and characterization of the prepared membrane are investigated. Moreover, cellulose acetate (CA) has been utilized on a large scale to separate oil/water systems; this is a biodegradable, eco-friendly, and abundant resource [152]. Thus, cellulose acetate (CA) nanofiber mats were prepared via electrospinning, and then deacetylated to form the d-CA nanofiber membranes, which are superhydrophilic in oil, oleophobic in water, and superamphiphilic in air. A multifunctional d-CA nanofiber filter can be applied as a water-separator nanomaterial for use in oil/water blends, emulsified oil/water, and oil/corrosive aqueous media under gravity. The d-CA nanofiber membranes show the largest separation flux and highest separation ability under the force of gravity. The separation flux was much higher here than in commercial CA (c-CA) membranes. The membranes showed good anti-pollution and self-cleaning capacities, with high cyclic stability and reusability [152].

On the other hand, higher-performance renewable nanomaterials with lower or even net zero carbon emissions are the main goal in the context of the desired sustainable and eco-friendly future. Therefore, various natural materials are considered promising candidates to be applied in the water purification processes. Table 4 presents some of the most recently fabricated sustainable nanofibers (eco-friendly) used in water purification processes [63,113,135,139,152,155,156,157,158,159,160,161,162,163,164,165,166,167]. Cellulose, chitosan, and silica are examples of natural materials that are attracting extensive interest due to their promising inherent, tunable, and biocompatible characteristics, in addition to their renewable nature [168].

## 6. Conclusions and Outlook

This review has surveyed the recent progress made in the fabrication of different forms of electrospun nanofibers and their applications in wastewater treatment. It concludes that these fabricated forms of nanofibers, especially the electrospun nanofibers utilized in the purification techniques, embody the concept of sustainable development.

In this review, many works demonstrate that electrospun nanofibers and their functionalized forms have been widely utilized to treat wastewater from various pollutants, such as organic and inorganic pollutants and oil/water separation, and they have illustrated considerable effects.

Due to the unique characteristics of nanofibers, they are considered a promising novel class of nanomaterials useful for diverse applications, such as water purification. Various methods have been investigated for the manufacturing of submicron polymeric nanofibers, such as nanolithography, self-assembly, multi-component spinning, flash spinning, electrospinning, etc. [54]. It can be easily concluded that electrospinning processes are extraordinary versatile in fabricating wide ranges of polymeric nanofiber nanomaterials, compared with other conventional techniques, and they can be considered an effective and multifunctional way to purify water without using any large infrastructure.

Despite the great efforts made to develop and improve upon the unique structures and functions of the nanofibers used for wastewater treatment and oil/water separation, many limitations must still be addressed. The properties of the polymer, composite, and nanofiber membranes are significantly affected by the media’s chemical and physical properties and the surrounding environment. Therefore, the properties of the fabricated nanofibers, such as their eco-friendly, degradability, renewability, non-toxicity, mechanical and thermal properties, as well as their availability, long-term durability, and cost-effectiveness, should be considered and further optimized to eliminate the other disadvantages. Additionally, the adhesion of pollutants, microorganism contaminations, biofilm formation, and eventual fouling during treatment processes are other serious challenges facing wastewater treatment using the various multifunctional electrospun nanofiber materials. Therefore, strategies are needed to develop and synthesize novel nanofiber materials with specific characteristics and self-cleaning features. Additionally, further efforts should be made to improve the surface-coating and impregnation methods, in order to reduce the fouling of membranes using suitable nanomaterials and to anchor the nanomaterials onto the surfaces of nanofiber materials. Additionally, the investigation of further techniques for the separation and retention of suspended nanomaterials after treatment is required to decrease the overall costs. More attention must still be paid to the fabrication of novel and renewable nanomaterials and functionalized nanofibers with specific properties and mechanical characteristics, with lower or even net zero carbon emissions, and with powerful capabilities in the purification of various pollutants from wastewater.

## Figures and Tables

**Table 1:** Summary of optimum conditions for nanofibers used in the removal of organic and inorganic pollutants.

NF and NFC	Contact Angle	Stress (MPa)	Elongation at Break (%)	Surface Area (m^2^ g^−1^)	Pore Volume (cm^3^ g^−1^)	Pore Diameter (nm)	Pollutant	Q_e_ (mg g^−1^)	%R	Ref.
PA/PAN-eTFC	32.3 ± 1.3°	13 ± 0.77	68 ± 0.28				TC		99.99	[55]
poly-CD	0	0.67 ± 0.06	2.50 ± 0.50	6.45	0.16	85.60	MB	124.102		[56]
DA@PDA	0			15.66			MB	88.2		[57]
Phytic acid doped polyaniline				33	9.6154 × 10^−2^	11.7	MB	43.4		[58]
PMMA-rGO	41			16			MB	698.51		[59]
PN-CSN				364	0.18		MB	400		[60]
PVA/S		3.47 ± 0.10	19.44 ± 0.28	24.72	0.0421	17.36	MB	400		[61]
CA/PEO/B							MB		93	[62]
ZIF-8@CS/PVA-ENF							MG	1000		[63]
Mo_2_N/MoO_2_							RhB			[64]
FAU/PLA							RhB MB		90	[65]
PU/10GO	0	11.94 ± 1.52 N/mm^2^	109.48 ± 3.66				RhB MB	77.15 109.88		[66]
ZIF-8/PAN							MB MG	120.84 1531.94		[67]
PCL/PEO@PDA				9.0741	0.008920	27.1665	MB MO	14.8 60.0		[69]
CA–GO/TiO_2_–NH_2_							IC MB	99.8 98.3		[70]
SPES	105	2.05 ± 0.18	13.14				MB Pb (II)	6.6 6.4		[71]
zirconia fibers							Allura Red dye	0.895		[72]
APAN/mMnp							IC	154.5		[73]
pTSA-PANI/PLLA	18			8.3 ± 0.4			MO	333		[74]
PMETAC/PES	0			20.51	0.1347	26.28	CR	208		[75]
p-ECNFs				144.70		15.7	CBB	141		[76]
PCSCN							DR80	322		[77]
NFMs-NH_3_^+^	0						MO SDS	406.6 636		[78]
ZnO-HT-PAN_H	95	7.86 + 3.21	19.89 + 2.25				RB19 RR195	276.36 245.76		[79]
TiO_2_/NCNF				56.7			MB		90.64	[80]
TiO_2_@CFs	130						MB		87.28	[81]
RC-NF@500 FC-NF@500				41 24			MB		97 79	[82]
TiO_2_-coated PEI							humic acid MB		~80 85	[83]
PES/TiO_2_	117		18.4 ± 0.2			199	Phenol		43	[84]
Ag@ZnO/TiO_2_		534 kPa		32.33	0.065		TC		91.6%	[85]
RGO/TiO_2_/PANCMA				28.4044	0.062028	11.7095	MG LMG		93.1 97.2	[86]
Cu_2_O/PLA							MO		92.9	[87]
PVA/PAA/MXene@PdNPs				13.48		27.35	4-NP 2-NA		94 90	[88]
Au@TiO_2_/NF				14		10–50	4-NP CR			[89]
Ag/Fe-EDTA-EDA-PAN NFs							MO		>96	[91]
PA/PEI-laccase							bisphenol A		90%	[92]

**Table 2:** Summary of optimum conditions for nanofibres used in the removal of inorganic pollutants.

NF and NFC	Contact Angle	Stress (MPa)	Elongation at Break	Surface Area (m^2^ g^−1^)	Pore Volume (cm^3^ g^−1^)	Pore Diameter (nm)	Pollutant	Q_e_ (mg g^−1^)	%R	Ref.
PVA/SHMP HENF				7.569	0.010632	12.15	La^3+^ Tb^3+^ Nd^3+^	181.82 243.90 217.39		[93]
PAN/Cyanex 272							Y(III) Eu(III)	200 400		[7]
PU/phytic acid	79.23		73.2				Pb^2+^	136.52		[100]
HG@NF							Pb^2+^	146.21		[101]
ASTPNM-15	149.0 ± 1.6			376	0.19		Pb^2+^	142.86		[102]
CS@PLLA							Cu^2+^	111.66		[8]
Polyethersulfone-poly (dimethyl amino) ethyl methacrylate nanofibrous							Cu^2+^	161.30		[103]
hordein/MBA/β-CD							Cu^2+^	88.50		[104]
T-Cg	0						Cu^2+^	399.14		[9]
PAA-SA NFHs							Cu^2+^	591.70		[105]
PAN/boehmite							Cd^2+^		74%	[106]
CS/PNC							Cd^2+^	232.55		[107]
PVA/SA							Cd^2+^	93.163		[108]
CA/Fe-MNZ							Ni^2+^	7.46		[109]
PVA (GA vapors) PVA (solution method)	69 39	11.57 17.61					Pb^2+^ Cu^2+^	161.7 58.3		[110]
PAA/dextran-polyaniline				168	19.210		Pb^2+^ Cu^2+^	1111.11 833.33		[111]
MWCNT-PEI/PAN	40.2°	11.05	3.59 ± 0.3				Pb^2+^ Cu^2+^	232.7 112.5		[112]
PVA/Chi							Pb^2+^ Cd^2+^	266.12 148.79		[113]
Modified PAN/PANI-nylon	4.5	37.4	17.2	57.77	6.607		Pb^2+^ Cd^2+^	960 911.72		[114]
PAN/CS/UiO-66-NH_2_							Pb^2+^ Cd^2+^ Cr^6+^	441.2 415.6 372.6		[115]
(CA/REC-SCV)_3.5_	0	1.12		2.5			Zn^2+^ Cd^2+^	104.31 99.33		[116]
SSC/TiO_2_/ZnO							Ni^2+^ Cu^2+^	282.3 298.1		[117]
CNFs/TiO_2_-PAN	20	3.93	0.23				Pb^2+^ Cu^2+^ Cd^2+^		87% 73% 66%	[118]
TPC-CNF		12.43					Cu^2+^ Ca^2+^ Pb^2+^	92.23, 97.34 82.19		[119]
P-PAN							Cu^2+^ Ni^2+^ Cd^2+^ Ag^+^	92.1 68.3 14.8 51.7		[120]
a-CNFs							Ca^2+^ Mg^2+^	57.66 65.55		[122]
ABS/PAN–ZnO	55						Cr^6+^		80%	[125]
(ZVI and CeO_2_) NF							Cr^6+^		≥96	[126]
PVAm-g-PVA/PAN membrane						838 ± 141/190 ± 33	Cr^6+^	133		[127]
PAN n-fib@Mat, PAN-ZnO_n_-fib@Mat, PAN/ZnO-TiO_2_ ^n-^fib@Mat							Cr^6+^	153.85 234.52 333.43		[128]
PAN/GO/ZnO		6.84 + 0.91					Cr^6+^	690		[130]
CS/PAAS							Cr^6+^	78.92		[131]
CSN-La							As^5+^	83.6		[132]
PAN/α-Fe_2_O_3_	30			10.37	0.034	11.56	As^5+^			[133]
0.05MNPs-G@PVA		7.20 ± 0.29	60.92 ± 10.53				Se(IV) Cr(VI)			[134]
CA/chitosan/SWCNT/Fe_3_O_4_/TiO_2_							Cr(VI), As(V)	345.2 285.6		[135]
chitosan-g-PNVCL/ZIF-8							Cr(VI) As(V)	495.6 439.5		[136]
PCL/CNF		2.4					Iron chromium		75% 99%	[138]
CS-PGMA-PEI							Cr (VI), Cu(II) Co(II)	138.96, 69.27 68.31		[139]

**Table 3:** Summary of optimum conditions for nanofibers used in oil/water separation.

NF	Water Contact Angle	Oil Contact Angle	Permeation Flux (m^−2^ h^−1^)	Intrusion Pressure (Pa)	Separation Performance (%)	Ref.
PLA/SiO_2_			17,800 L		100	[141]
PLA/ZIF-8@C600					72.59	[142]
PDMS/TA-Al^3+^/PI	153.64 ± 1.6°	0	6935	gravity-driven	99	[143]
ZnO/PDMS-PI	120	0	3706 ± 161	gravity-driven	99	[144]
PVDF/rGO/TiO_2_	100				98.46	[145]
[ABS]/ZnO	138	0		gravity-driven		[146]
Au@ZIF-8@PAN-TD	155.5	0	<200	gravity-driven	97.8%	[147]
PSBMA/PES	0	150 (UWOCAs)	3723	gravity-driven	99%	[148]
CNTs-PVA	32 ± 6°		60	20 k	100	[149]
SPAN-PPy/ZnO	0	>150		>120 k	96	[151]
d-CA	0	0	38,000	gravity-driven	99.97	[152]
SSA-PAAS-g-PVDF	0		53,574	>6000		[153]
PAA/NFM	27	>128	5142	gravity-driven	>97.2	[154]

**Table 4:** Some of recent fabricated sustainable nanofibers (eco-friendly) used in water purification processes.

Nanofiber	Pollutant	Adsorption Capacity (mg/g)	Oil/Water Separation Efficiency	Photocatalytic Degradation Efficiency	Ref.
Cellulose nanofibers and calcium alginate beads	Cu (II)	56.50			[155]
Chi/PEO permutit electrospun nanofibers	Cr(VI)	208			[156]
Oxidized regenerated cellulose nanofiber membrane	Cu (II), Pb (II)	20.78 and 206.1			[157]
Chi/g-C_3_ N_4_/TiO_2_	Cr(VI)	165.3			[158]
Electrospun chitosan–polyethylene oxide-oxidized cellulose biobased composite	Cu	15.72			[159]
Nitro-oxidized carboxy-cellulose nanofibers obtained from plants	Hg	257.07			[160]
PVA/Chi	Pb (II)	266.12			[113]
Chi-PGMA-PEI	Cr (VI) Cu (II) Co (II)	138.96 69.27 68.31			[139]
Cellulose acetate/Chi/SWCNT/Fe_3_ O_4_/TiO_2_	Cr (VI) As (V)	345.2 285.6			[135]
ZIF-8@Chi/PVA	Malachite green (MG)	1000			[63]
Chi/sodium alginate	Acid Black-172 Methylene blue	817.0 1488.1			[161]
Cellulose nanofibers	Acid green 25	683			[162]
Cellulose nanofibers	MB	502			[163]
Carbon dots/cellulose nanofibers	MB			99.9	[164]
Cellulose nanofibers	Oil phase		99.97		[152]
Cellulose acetate/cellulose fiber paper composite membrane	Oil phase		84		[165]
PVDF-SiO_2_ nanofibers membrane	Oil phase		99		[166]
poly(ethylene-co-polyvinyl alcohol) (EVOH) nanofiber membranes	Oil phase		99.9		[167]

## Data Availability

Data are contained within the article.

## Abbreviations

CANFs—cellulose acetate nanofibers; Chi—chitosan; CMCNF—carboxymethyl cellulose nanofibril; CNT—carbon nanotubes; DA—cellulose acetate; DPANI—dedoped polyaniline; GO—graphene oxide; HENF—hydrogel electrospun nanofiber; MB—methylene blue; MBA—N,N′-methylene bisacrylamide; PA—poly amide; PAA—polyacrylic acid; PAN—polyacrylonitrile; PCL—polycaprolactone; PDM—poly (dimethylsiloxane); PEI—polyetherimide; PEO—polyethylene oxide; PES—polyethersulfone; PGMA—poly (glycidyl methacrylate); PLLA—poly(L-lactic acid); PANI—polyaniline; PMMA—poly methyl metacrylate; poly-CD—polycyclodextrin; PPy—polypyrrole; PSBMA—poly-(sulfobetaine-methacrylate); PU—polyurethane; PVA/SHMP—poly (vinyl alcohol)—sodium hexametaphosphate; PVDF—polyvinylidene fluoride; SDS—sodium dodecylsulfonate; SPAN—stabilized polyacrylonitrile; SPAN-PPy—stabilized polyacrylonitrile-polypyrrole; STPNM—superamphiphilic (SiO_2_/TiO_2_) porous nanofibers membranes; SVA—solvent vapor annealing; TFNC—nanofibrous nanocomposite; ZVI—zerovalent iron.
